# Translating Translation to Mechanisms of Cardiac Hypertrophy

**DOI:** 10.3390/jcdd7010009

**Published:** 2020-03-10

**Authors:** Michael J. Zeitz, James W. Smyth

**Affiliations:** 1 Fralin Biomedical Research Institute at Virginia Tech Carilion, Roanoke, VA 24016, USA; mjzeitz@vtc.vt.edu; 2 Department of Biological Sciences, College of Science, Virginia Tech, Blacksburg, VA 24061, USA; 3 Department of Basic Science Education, Virginia Tech Carilion School of Medicine, Roanoke, VA 24016, USA

**Keywords:** translation, hypertrophy, connexin43, mTOR

## Abstract

Cardiac hypertrophy in response to chronic pathological stress is a common feature occurring with many forms of heart disease. This pathological hypertrophic growth increases the risk for arrhythmias and subsequent heart failure. While several factors promoting cardiac hypertrophy are known, the molecular mechanisms governing the progression to heart failure are incompletely understood. Recent studies on altered translational regulation during pathological cardiac hypertrophy are contributing to our understanding of disease progression. In this brief review, we describe how the translational machinery is modulated for enhanced global and transcript selective protein synthesis, and how alternative modes of translation contribute to the disease state. Attempts at controlling translational output through targeting of mTOR and its regulatory components are detailed, as well as recently emerging targets for pre-clinical investigation.

## 1. Introduction

Upon terminal differentiation, the majority of cardiomyocytes lose their ability to proliferate shortly after birth. Postnatally, heart growth is predominantly due to an increase in cardiomyocyte size, designated hypertrophic growth [1]. In adults, hypertrophic growth is an adaptive mechanism in response to a need for increased cardiac output. When physiological, as occurs during periods of intense exercise training, this growth in heart size can be beneficial and allows adaptation to increased demand. In contrast, cardiac growth arising from pathological stress results in remodeling, decreased pumping efficiency, and typically progresses to heart failure [2,3]. At the cell and molecular level, pathological hypertrophy is characterized by a global increase in protein synthesis, re-activation of fetal genes, and fibrosis. While a great deal is known about the underlying stimuli and pathological remodeling associated with cardiac hypertrophy, new details are still emerging regarding the molecular mechanisms governing regulation of protein translation necessary for cellular hypertrophic growth, and their role in the progression to heart failure.

Translational regulation is increasingly recognized as a means for tissue to rapidly respond to environmental stress. This has direct implications for identifying molecular markers of disease where mRNA abundance has historically served as a proxy for protein expression. Importantly, there are increasing reports of frequent and significant disconnects between levels of mRNA transcripts and associated proteome (reviewed in [4]). While many factors contribute to total protein abundance, including transcript stability and protein degradation rates; high throughput RNA sequencing combined with proteomic analysis has demonstrated that translational control is the primary determinant, and is a better predictor of protein abundance than transcript levels [5]. Recent integration of multi omics experiments on normal hearts and hearts from dilated cardiomyopathy patients highlight the complex diversity of translational mechanisms governing cardiac homeostasis, and further reveal discordance among transcript and protein levels during dilated cardiomyopathy, suggesting a role in disease progression [6]. As pathological cardiac hypertrophy requires widespread alterations to the normal cardiomyocyte proteome, it is crucial to understand how altered translational regulation contributes to the diseased state. 

Protein translation occurs in three main phases, initiation, elongation, and termination. Of these three phases, translation initiation is the most tightly regulated, and is considered the rate-limiting step of protein synthesis (reviewed in [7]). Canonical eukaryotic translation initiation employs a ribosome scanning mechanism to identify an AUG start codon [8]. This cap-dependent mechanism of translation initiation comprises several well defined steps (reviewed in [9]). Briefly, the small (40S) ribosomal subunit binds the eIF2-GTP-Met-tRNAi ternary complex and eukaryotic initiation factors eIF1, eIF1A, and eIF3 to form the 43S preinitiation complex (PIC). The PIC is subsequently recruited to the 7-methylguanosine cap of the RNA transcript by the eukaryotic initiation factor 4F complex (eIF4F). The heterotrimeric eIF4F complex consists of the m^7^G cap-binding protein eIF4E, the RNA helicase eiF4A, which unwinds secondary structure in the 5′ untranslated region (UTR), and the scaffold protein eIF4G. Interaction between the mRNA poly(A) tail and eIF4G is mediated by poly(A) binding protein C1 (PABPC1), and serves to circularize the transcript [10]. The PIC then scans the 5′UTR until it reaches an AUG in a favorable context for translation initiation [11]. Numerous points along the process of translation initiation are targets for regulation both on a global scale and for select transcripts. Components of translational regulation whose functions are involved in pathological stress in cardiac hypertrophy and progression to heart failure are discussed below.

## 2. Global Regulators of Translation

### 2.1. eIF4E

The initiation factor eIF4E plays a critical role in regulating cap-dependent translation. Early studies on eIF4E expression noted its low abundance relative to other initiation factors that had been identified at the time, leading to the idea that its cap binding function within the eIF4F complex is rate-limiting for translation initiation [12,13]. It is clear that modulation of eIF4E levels or activity can enhance translation efficiency for a subset of mRNA transcripts [14]. Sensitivity to eIF4E for a given transcript is often correlated to its degree of 5′UTR secondary structure. Transcripts with complex 5′UTR regions are hypothesized to be weakly translated and thus more dependent upon eIF4E [15,16]. This may be attributed to the ability of eIF4E to stimulate eIF4A helicase activity, independent of cap binding [17,18]. These eIF4E sensitive transcripts typically encode proteins with roles in cellular proliferation and survival. Thus, levels of eIF4E are important for regulating normal cell growth, and its dysregulation is observed to play a role in both cardiac hypertrophy and cancer, where increased protein synthesis is required [19,20]. When overexpressed, eIF4E activity contributes to oncogenic transformation, and has been implicated in poor survival in numerous malignancies (reviewed in [21]). Regulation of eIF4E activity is also modulated by phosphorylation [22]. The kinases known to be responsible for eIF4E phosphorylation are the mitogen-activated protein kinase (MAPK) interacting protein kinases 1 and 2 (Mnk1/2), which are activated by the MAPKs p38 and ERK pathways [23,24]. Knockout mouse models of Mnk1/2 have revealed that phosphorylation of eIF4E is not required for global protein synthesis under basal or serum-stimulated conditions, exposing the possibility of transcript specific effects in eIF4E stimulated translation [23]. In agreement with the idea of such eIF4E sensitive transcripts, Mnk mediated phosphorylation of eIF4E was found to facilitate selective translation of RNA with a hairpin structure in the 5′UTR in a cell free translation system [25]. Just as observed with overexpression of eIF4E, increased levels of eIF4E phosphorylation are correlated with oncogenesis [26].

Adult cardiomyocytes are terminally differentiated, and translation rates in the adult heart are relatively low in comparison to other tissues [27]. Translational control in the heart is rapidly altered in response to a number of growth inducing stimuli. Studies have observed enhancement of both global and transcript specific translation in mouse and rat hearts undergoing hypertrophic growth [28,29,30]. In line with its role in cellular growth control, increased eIF4E expression and phosphorylation are associated with progression of cardiac hypertrophy. In a mouse model of chronic hypoxia, eIF4E RNA and protein levels increase in cardiac tissue along with markers of hypertrophy [31]. It has long been observed that experimental pressure overload amplifies protein synthesis through increases in both translation rate and/or capacity [32,33,34]. In a canine model of pressure overload induced hypertrophy, eIF4E phosphorylation was significantly elevated during both acute and chronic phases [34]. Following acute pressure overload in mice by transverse aortic constriction (TAC), a screen of candidate genes involved in cardiac hypertrophy revealed transcript-specific increases in translational activity, measured by increases in polysome association [28]. Transcripts with increased translation, with the exception of c-myc, had no significant increase in transcript abundance, further highlighting the importance of translational regulation. In addition to being involved in cardiac growth, these candidate transcripts were selected due to the presence of high secondary structure in their 5′UTR, implicating a role for eIF4E stimulation of eIF4A in the enhanced translation of these putative weakly translated transcripts.

### 2.2. mTORC1

**Figure 1:**
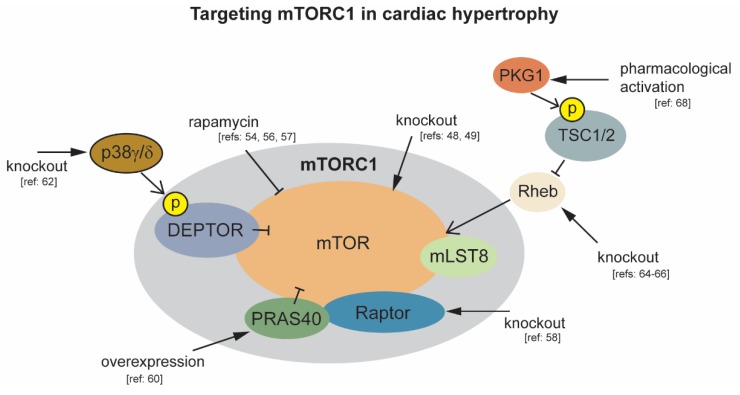
Modulation of mTORC1 activity to protect against cardiac hypertrophy. Black arrowheads indicate experimental interventions targeting mTORC1 and its regulatory components.

A key regulator of cellular growth is the mechanistic target of rapamycin (mTOR). Extensive research has identified mTOR’s role in responding to intracellular and environmental signals to balance processes including translation and autophagy (reviewed in [35]). The mTOR protein is known to occur in two multiprotein complexes termed mTOR complex 1 (mTORC1) and 2 (mTORC2). In addition to mTOR, mTORC1 consists of regulatory components including proline-rich Akt substrate of 40 kDa (PRAS40), regulatory-associated protein of mTOR (Raptor), mammalian lethal with sec-13 protein 8 (mLST8), and DEP domain- containing mTOR-interacting protein (DEPTOR) [36,37,38,39,40] (Figure 1). One means by which mTORC1 exerts its effect on translation initiation is through phosphorylation of eIF4E-binding protein-1 (4E-BP1) [41]. 4E-BP1 inhibits translation initiation by interacting with eIF4E to prevent eIF4F complex formation [42]. In response to stimuli, mTORC1 phosphorylation of 4E-BP1 prevents its binding and sequestration of eIF4E, which results in induction of protein synthesis. mTORC1 also phosphorylates the ribosomal S6 kinase (S6K), which plays a role in translation initiation and ribosome biogenesis [43,44]. Importantly, cell size in mammals is controlled through mTORC1 regulation of both 4EBP1 and S6K [45]. In addition to mTORC1 and mTORC2 roles in cardiac development and homeostasis, mTORC2 contributes to cardiomyocyte survival in response to stress [46]. Given the vast amount of data describing mTORC1 in translational regulation, its role in cardiac hypertrophy and targeting for therapeutic intervention is discussed below.

mTORC1 is required for normal cardiac development and function, with cardiac-specific deletion of mTOR in mice resulting in death during embryogenesis, and when deletion is temporally induced during adulthood [47,48,49]. As mTORC1 activity is responsive to both physiological and pathological hypertrophic stimuli, including but not limited to testosterone, β-adrenergic receptor activators, atrial natriuretic peptide, IGF1, and pressure overload, a large number of animal studies have investigated the role of mTORC1 in the progression of cardiac hypertrophy [50,51,52,53,54]. Comparison of physiological versus pathological cardiac hypertrophy in mice reveals an increase in mTOR activity following 6 weeks of exercise training resulting in physiological growth. An opposing effect of decreased mTOR activity accompanied with a decline in cardiac function is observed following 8 weeks of chronic pressure overload induced hypertrophy by TAC [55]. This supports the observation that the nature of the stress influences cardiac signaling to differentiate physiological vs. pathological hypertrophy [3]. Earlier studies report a rapid activation of mTOR in response to acute pressure overload. Inhibition of mTOR by pretreatment with rapamycin reduces markers of cardiac hypertrophy in this mouse model of ascending aortic constriction, including limiting increases in heart weight and myocyte size [56]. Subsequent work indicates that rapamycin inhibition of mTOR can partially restore normal heart size and function in mice with established cardiac hypertrophy. Following rapamycin treatment, mice subjected to ascending aortic constriction had improved heart weight to body weight ratios, and improved fractional shortening and ejection fractions [54]. Together, these studies suggest that an initial increase in mTOR activity, which may be beneficial, occurs in response to both pathological and physiological stimuli. As opposed to physiological growth, during sustained pathological stimuli, this compensatory hypertrophy progresses to pathological hypertrophy with cardiac dysfunction and subsequent heart failure. One drawback to chronic rapamycin treatment is that it can inhibit mTORC2 in some cell types, and rapamycin does not completely inhibit all mTORC1 functions [57]. For these reasons a more targeted approach was needed to assess the effect of inhibition of mTORC1 for therapeutic intervention.

One approach employed was to conditionally knock out raptor, an essential component of mTORC1, but not mTORC2, in cardiac tissue. Raptor deletion in adult cardiac tissue, under normal physiological conditions, causes a drop in ejection fraction at 38 days post deletion. When exposed to pressure overload, raptor knockout mice rapidly develop dilated cardiomyopathy within 1 week, and display no adaptive hypertrophic growth [58]. The deleterious effect of complete inhibition of mTORC1 activity by mTOR or raptor deletion, even under physiological conditions, limit its therapeutic potential in the heart. Alternatively, modulating the activity of mTORC1 regulatory components in mouse models of hypertrophy has demonstrated more encouraging results. One example is targeting of PRAS40, a regulatory component of mTORC1 that interacts with raptor to inhibit mTORC1 activity [59]. Phosphorylation of PRAS40 by AKT relieves inhibition of mTORC1. Overexpression of PRAS40 prior to TAC prevents hypertrophic growth. PRAS40 overexpression is also capable of stabilizing cardiac function in mice post initiation of TAC induced hypertrophy [60]. DEPTOR also functions as an mTORC1 inhibitor. Phosphorylation of DEPTOR by mTORC1 leads to DEPTOR degradation and increased mTORC1 activity [61]. It was recently observed that DEPTOR is also phosphorylated by the stress associated mitogen activated protein kinase p38 to promote its degradation and elevate mTOR activity. In wild type mice, activation of p38γ and p38δ isoforms by angiotensin II correlates with markers of cardiac hypertrophy including significant increases in left ventricular and whole heart mass, and elevation of cardiomyocyte area. Following the same treatment with angII, p38γ/δ knockout mice display no hypertrophic growth [62]. Ras homolog enriched in brain 1 (Rheb1) lies upstream of mTOR and downstream of Tuberous sclerosis proteins 1 and 2 (TSC1/2), and functions as an mTOR activator [63,64]. Rheb1 activation of mTOR is required for postnatal heart growth in mice as evidenced by significantly reduced heart weight 8–9 days postnatal, and subsequent heart failure following cardiac-specific deletion of Rheb [64,65]. In adult hearts, genetic deletion of Rheb1 in cardiomyocytes suppresses cardiac hypertrophy in response to pressure overload. Following TAC, cardiac specific Rheb1^–/–^ mice had lower heart weight, cardiomyocyte size, and fibrosis than control mice [66]. The TSC1/2 complex is also a major inhibitor of mTORC1 activity through inhibition of Rheb1 [67]. Recently, it was demonstrated that TSC2 phosphorylation by protein kinase G1 (PKG1) can prevent cardiac hypertrophy in response to pressure overload by suppressing mTORC1 signaling. Importantly, phosphorylation of PKG1 target sites in TSC2 does not affect the basal level of mTOR activity [68]. Together, these studies indicate that targeting of the specific regulatory components of mTORC1 may provide effective therapies while minimizing off target effects associated with inhibition of the mTORC kinase itself.

### 2.3. Endoplasmic Reticulum Stress and the Unfolded Protein Response

In mammals, the unfolded protein response (UPR) has evolved to maintain endoplasmic reticulum (ER) proteostasis, and determine cell fate through promotion of apoptosis under conditions of prolonged ER stress. The UPR comprises signal networks activated by stress sensors within the ER including inositol-requiring kinase 1 (IRE1α), dsRNA-activated protein kinase-like ER kinase (PERK), and activating transcription factor 6 (ATF6). Given the important role of the UPR in controlling ER function and global protein translation its dysregulation has been correlated with cardiovascular disease pathogenesis [reviewed in [69,70]]. Induction of cardiac hypertrophy in mouse models such as TAC have been demonstrated to induce ER stress and activate the UPR resulting in an increase in cardiomyocyte apoptosis preceding heart failure [71,72]. Blackwood et al. revealed that due to the increased requirements for protein synthesis and folding, ATF6 is activated in response to both physiological exercise and TAC induced hypertrophy in mice [73]. Under these conditions, cardiomyocyte specific deletion of ATF6 was found to impair protein synthesis and compensatory cardiac hypertrophy. Interestingly, ATF6 mediated cardiac growth induced *RHEB* leading to increased protein synthesis through activation of mTORC1, establishing a link between cardiac growth and the UPR. A recent study examining heart failure with preserved ejection fraction (HFpEF) further highlights the importance of the UPR in cardiovascular disease, where failure to activate IRE1α was detected in both a mouse model and clinical HFpEF samples [74].

### 2.4. PABPC1

During the initial steps of translation initiation, PABPC1-mediated circularization of mRNA serves to both stabilize transcripts and stimulate translation initiation [75,76]. While PABPC1 mRNA is present at similar levels in fetal and adult hearts, its protein levels are substantially reduced in both the mouse and human adult heart, correlating with low rates of protein synthesis. Recently, it was shown that this post-transcriptional reduction in PAPBC1 protein is due to shortening of its mRNA poly(A) tail to approximately 20 nucleotides in the adult heart. PABPC1 protein is upregulated in both physiological and pathological cardiac hypertrophy with concomitant increased protein synthesis correlated with an increase in its poly(A) tail length. Interestingly, experimental overexpression of PABPC1 in adult mouse hearts leads to hypertrophic growth, but does not induce expression of markers of pathological hypertrophy [27]. This suggests that increased translation rates alone are insufficient for the transition from hypertrophic growth to pathological remodeling and heart failure. Rather, current evidence indicates that the type of stress, pathological vs. physiological, determines the clinical presentation. For example, Perrino et al. compared intermittent pressure overload by TAC with consistent durations of exercise training to demonstrate that, in regards to cardiac hypertrophy, it is actually the pathological nature of a given stress that results in a cardiac dysfunction, as opposed to the duration of cardiac stress [3]. It was observed however, that the duration of stress in the form of chronic vs. intermittent pressure overload was a determinant of the degree of cardiac hypertrophy.

## 3. Local Regulatory Elements

### AU-Rich Element Binding Proteins

While the features of cardiac remodeling associated with cardiac hypertrophy and progression to heart failure have been extensively studied, much less is known about the alterations to translational regulation associated with these changes. A recent finding that sheds light on translational alterations during such progression is the upregulation of AU-Rich element (ARE) RNA-binding proteins in failing hearts. AREs are found in 5–8% of human mRNA transcripts, and are typically present in genes demonstrated to require tight regulation, such as those with a role in growth and inflammation [77]. Regulation of mRNA stability by AREs allows for rapid changes in protein levels. AREs, initially reported to function in mRNA destabilization [78], are now recognized to play either a stabilizing or destabilizing role depending on the function of the ARE-binding protein (reviewed in [79]).

A chronic immune inflammatory response with elevated cytokines is observed in congestive heart failure (reviewed in [80]). Elevation of inflammatory cytokines during cardiac hypertrophy has recently been observed to, at least partially, rely on translation of inflammatory genes mediated by enhanced stability of their mRNA by ARE-RNA binding proteins. The increase in ARE binding protein activity during the progression to heart failure is accompanied by their translocation from the nucleus to the cytoplasm [81,82]. One example of an ARE binding protein induced during human heart failure and in a mouse model of TAC induced pressure overload is brain-expressed X-linked protein 1 (BEX1). BEX1 has been implicated to play a role in numerous biological processes including cell cycle regulation, muscle regeneration, and to function as a tumor suppressor [83,84,85]. BEX1 induction during heart failure coincides with increased expression of proinflammatory genes such as tumor necrosis factor-α (TNFα). Although BEX1 was not found to bind AREs directly, complexes containing BEX1 bind and modulates the stability of transcripts encompassing AREs, which are often present in the 3′ UTR of inflammatory genes. Transgenic mice expressing BEX1 show a worsening of pathological features following TAC induced pressure overload, while *Bex1* gene deletion was protective [81]. Therefore, BEX1 associated RNA stability results in an increase in translation of proinflammatory genes, likely contributing to subsequent heart failure following pressure overload induced hypertrophy.

**Figure 2:**
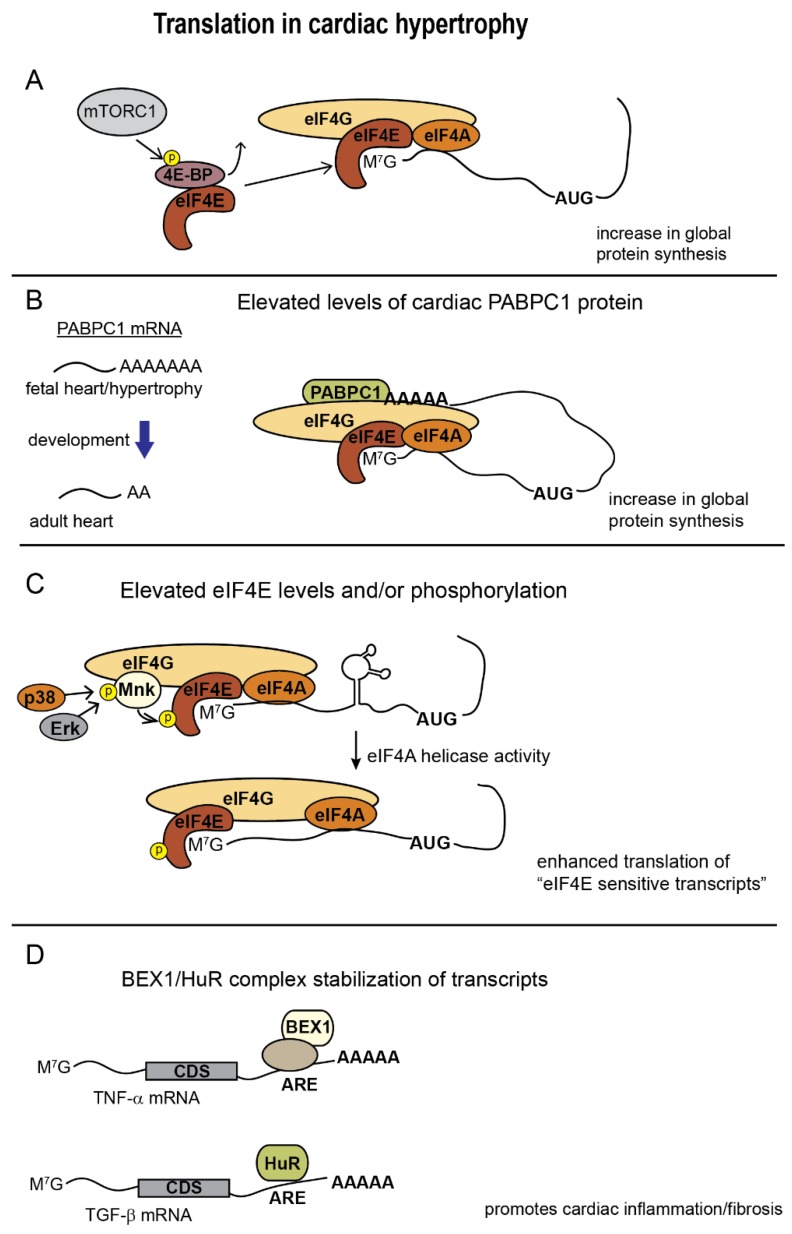
Changes in translation in cardiac tissue undergoing hypertrophy. (**A**), mTORC1 phosphorylation of 4E-BP releases eIF4E to increase global protein synthesis; (**B**), Elongation of PABPC1 poly(A) tail length in response to hypertrophic signaling elevates its translation in the adult heart and allows PABPC1 to enhance global protein synthesis; (**C**), MnK mediated phosphorylation of eIF4E in response to MAPK signaling stimulates eIF4A helicase activity to boost translation of 5′UTRs with complex secondary structure.; (**D**), HuR, and BEX1 (in combination with other proteins) bind AU-rich elements (ARE) during heart failure and promote translation of fibrosis and proinflammatory genes.

Another ARE binding protein upregulated in failing hearts is human antigen R (HuR) [82]. HuR is a ubiquitously expressed member of the embryonic lethality and abnormal visual system (ELAV) protein family [86]. Investigation of HuR in neonatal rat ventricular myocyte culture found its activation and translocation from the nucleus to the cytoplasm in response to phenylephrine induced hypertrophic signaling. HuR knockdown or pharmacological inhibition prevents hypertrophic growth, while its overexpression induces hypertrophy, indicating both necessity and sufficiency in hypertrophy of rat myocytes [87]. Interestingly, HuR is capable of stabilizing eIF4E mRNA resulting in increased eIF4E protein, and thus linking its activity with increased translation [88]. HuR is also upregulated in a mouse model of TAC induced hypertrophy where it was found to mediate cardiac fibrosis likely through stabilization of TGF-β transcripts (Figure 2). HuR activity is negligible in normal adult hearts, and thus cardiomyocyte specific deletion does not affect basal cardiac function, yet HuR deletion does reduce hypertrophy and cardiac functional decline in response to TAC. Importantly, the authors report clinical benefits from pharmacological HuR inhibition even when hypertrophy is already detectable [82].

## 4. Alternative Mechanisms of Translation Initiation in Cardiac Biology

In addition to ‘canonical’ translation, alternative mechanisms of translation initiation are increasingly recognized as contributing to the regulation of protein expression. Such alternative translation initiation can regulate translational efficiency through expression of upstream open reading frames (uORFs), alternate modes of m^7^G cap recognition, and cap-independent translation. Translation initiation occurring within a protein coding sequence is capable of generating functional truncated protein isoforms, as has been observed to occur with *GJA1* and *MAVS* mRNA, for example (reviewed in [89]). Modulation of translation and employment of alternative translational mechanisms are hallmarks of the cellular response to multiple forms of stress and is clearly relevant to cardiovascular disease. One example is the inhibition of global protein synthesis and the translational upregulation of select transcripts occurring during the integrated stress response [90,91,92]. As more eukaryotic mRNAs are identified which undergo alternative translation, their impact on heart disease and pathological hypertrophy needs to be considered. Below, we focus on *GJA1* mRNA and the relationship between regulation of alternative translation initiation and electrical coupling in the heart.

### 4.1. Altered Connexin43 Protein Translation in Heart Disease 

Cardiac stress also influences levels of Cx43, the predominant connexin of the ventricular myocardium, by modulating its translation [93,94]. How altered translational regulation of *GJA1*, encoding connexin43 (Cx43), may affect cardiac health is discussed below. Cx43 gap junctions at intercalated discs enable the flow ions between cardiomyocytes to facilitate cardiac conduction and rhythmic contraction. Alterations in cardiac Cx43 levels and gap junction function are understood to underlie increased propensity for arrhythmias [95,96,97]. Remodeling of intercalated disc structure is reported in a number of heart diseases including pathological cardiac hypertrophy [98]. Specifically, during pathological cardiac hypertrophy, a progressive reduction in Cx43 immunofluorescence signal at intercalated discs occurs [99,100]. Treatment with isoprenaline, a beta-adrenergic receptor agonist used to induce cardiac hypertrophy, activates p38, JNK, and ERK map kinases within 24 h leading to AP1 mediated transcription. This correlates with increases in Cx43 mRNA and protein expression [101]. Studies using human biopsies have found higher levels of Cx43 in patients with hypertrophic cardiomyopathy where increased lateralization of Cx43 protein away from intercalated discs is reported. This is a distinct from significantly reduced levels of Cx43 throughout the cardiomyocyte in dilated cardiomyopathy [101]. Similar results were observed in biopsies from patients with aortic stenosis and compensated or decompensated cardiac hypertrophy. In these patients, increased Cx43 expression and lateralization was observed in hypertrophy with preserved ejection fraction, while patients with decompensated ejection fraction had reduced Cx43 expression [100]. In a rat model of compensated hypertrophy, elevated levels of heterogeneously localized Cx43 is associated with increased ventricular tachycardia [102]. Together, these data reveal an initial increase in Cx43 levels, albeit with an accompanying increase in lateralization away from the intercalated disc, during compensatory cardiac hypertrophy followed by a significant decrease in Cx43 expression in failing hearts. A recent study implicates CUGBP Elav-like family member 1 (CELF1) mediated Cx43 mRNA degradation in mouse models with dilated cardiomyopathy suggesting a mechanism for reduced Cx43 protein levels mentioned above [103]. Lateralization and/or reduced expression of Cx43 can both result in arrhythmias of sudden cardiac death, highlighting the importance of maintaining proper Cx43 expression in cardiac tissue and maintenance of Cx43 at the intercalated disc [104]. Recent work has revealed mechanisms that regulate Cx43 translation and is enhancing our understanding of Cx43 gap junction formation in cardiac tissue.

**Figure 3:**
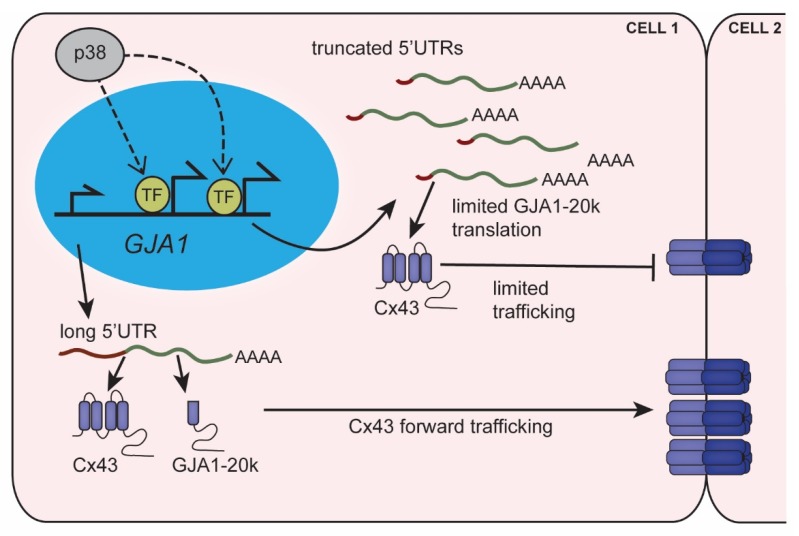
***GJA1*-5′UTR truncation regulates alternative translation limiting gap junction formation.** Stress signaling in the heart activates p38, which mediates transcription at alternate *GJA1* promoters resulting in truncated 5′UTRs. Truncated *GJA1*-5′UTRs favor translation of Cx43 over its internal translation product GJA1-20k. Reduced GJA1-20k levels lead to suppression of Cx43 trafficking and limits gap junctions at intercellular junctions.

Cx43 encoding *GJA1* mRNA is subject to alternative translation initiation resulting in the generation of n-terminally truncated protein isoforms. The most prominent *GJA1* internal translation product is a 20 kDa protein termed GJA1-20k [105,106,107]. We have demonstrated a role for GJA1-20k in promoting Cx43 oligomerization to facilitate gap junction formation in epithelial cells using a model of TGF-β induced EMT. TGF-β stimulation limits internal translation of GJA1-20k while promoting Cx43 expression resulting in gap junction dissolution [108]. Alternative translation of *GJA1* is also affected by cellular stress such as hypoxia/ischemia, and elevated levels of the MAPKs p38 and ERK [93]. An increase in GJA1-20k is observed in brain and heart tissue subject to ischemia [106,109]. In the heart, ischemia leads to a reduction in Cx43 levels at the intercalated disc. Exogenous gene delivery of GJA1-20k to hearts prior to no flow ischemia maintains Cx43 localization at intercalated discs [110]. Using this model, it was demonstrated that GJA1-20k could also protect against damage from ischemia/reperfusion [109]. We recently found that chronic hypoxic stress in mouse and human cell lines, as well as human iPSC derived cardiomyocytes (HiPSC-CMs), reduces levels of GJA1-20k, and this is correlated with a reduction in Cx43 gap junction formation [93]. Thus, the potential protective role of GJA1-20k in cardiomyopathy lends urgency to elucidating the *cis* and *trans* acting factors governing *GJA1* translation. The *Gja1*-5′UTR is the only region of *Gja1* mRNA with sequence variability. In mice, *Gja1*-5′UTR variants are generated through alternate promoter usage and splicing, and function to modulate translation of full length Cx43 protein [111]. Using rapid amplification of cDNA ends (RACE) we demonstrated the role of *GJA1*-5′UTR variants in governing *GJA1* translation in mouse and human cells, including HiPSC-CMs [93]. TGF-β is induced during cardiac hypertrophy and TGF-β signaling through TGF-β activated kinase (TAK1) stimulates MAPK activity to modulate gene expression [112,113]. In cardiac muscle subject to pressure overload, TAK1 and p38 activity are significantly upregulated [114,115]. Using specific inhibitors, we have found that *GJA1*-5′UTR selection is dynamically regulated by p38 signaling downstream of TGF-β. Upon p38 activation, there is a shortening of *GJA1*-5′UTR length arising from alternate transcription start site (TSS) usage. This 5′UTR shortening correlates with altered GJA1-20k translation where transfection of these 5′ UTR variants into *GJA1*^–/–^; cells is sufficient to phenocopy effects on *GJA1* translation, shutting down GJA1-20k just as we observed in TGF-β/hypoxia studies. These findings confirm a role for *cis* elements within the *GJA1*-5′UTR in regulation of GJA1-20k levels with further research necessary on understanding mechanisms which may include specific *trans*-acting factors such as RNA binding proteins. p38 activity is reported to increase with age, which may induce a state of chronic stress signaling [116,117]. We have found elevated p38 in aged cardiac tissue correlating with an increase in truncated UTR expression, reduced GJA1-20k, and reduced Cx43 at intercalated discs [93] (Figure 3). Together, these data link age-related alteration of translational regulation to cardiac decline and subcellular pathological remodeling affecting electrophysiology.

### 4.2. Translation as a Regulator of Cardiac Ion Channel Function

Alternative translation is increasingly recognized to play an important role in cardiac function, with novel translation products still being discovered [6]. In addition to Cx43, alternative translation of other ion channels has been reported. Potassium channel genes *TREK-1* (K_2P_2.1) and *TREK-2* (K_2P_10.1), which are expressed in cardiac tissue, undergo alternative translation yielding n-terminally truncated protein isoforms [118,119]. Interestingly, these shorter K_2P_ alternative translation products result in functional changes in conductance and display altered drug sensitivity [120,121]. Calcium channel subunits generated from the ORAI transcript are also generated through alternative translation [122,123]. Indeed, many cardiac proteins from ion channels to sarcomeric proteins are relatively large, and biochemical analysis has historically proven difficult. Additional isoforms on western blots are often, and appropriately, attributed to splice-variants and cleavage products, but we must now include alternative translation initiation as a source of such biologically active polypeptides. Autoregulation of full-length counterparts in facilitation of trafficking or ion channel function by internal translation products is just one facet of how this may impact disease. This is highlighted through the ability of GJA1-20k to ‘moonlight’ in regulation at the mitochondria [109,124], and that both GJA1-20k and the cleaved Ca_V_1.2 C-terminus can impact transcription [125,126]. Regarding regulation, our findings on dynamic *GJA1* mRNA UTR usage discussed above are unlikely relevant to just one gene, and it is our hope that they will inform studies identifying global shunting of translation through similar mechanisms across many mRNAs impacting cardiac function. It remains to be seen what role these polypeptides play in maintaining normal cardiac function and how other non-ion channel mRNAs also subject to alternative translation initiation contribute to such pathologies.

## 5. Future Perspectives

Technological innovations in translation research are allowing for a global picture of post transcriptional regulation of gene expression in cardiac tissue during normal and disease states to emerge. The heart is a complex tissue consisting of multiple cell types that change during disease progression. The creation of a Cre recombinase inducible RiboTag mouse line has enabled analysis of cell-type specific translational regulation of mRNA transcripts from complex heterogeneous tissue [127]. Combining ribosome tagging with ribosome profiling is allowing for a comprehensive snapshot of cell-type specific translational activity [128,129]. Application of this technology to TAC operated mice elucidated the timing and composition of cardiomyocyte specific translationally regulated genes and highlighted an overall repressive effect of uORFs on translation during cardiac hypertrophy [130]. Another form of post transcriptional gene regulation involves the most abundant mRNA modification, which consists of methylated adenosine at position 6 (N^6^-methyladenosine, m^6^A) [131,132]. Several recent studies implementing m^6^A-seq have uncovered the dynamic nature of global m^6^A levels in cardiac tissue under stress and during disease [133,134,135]. These studies identified an increase in m^6^A mRNA levels in failing human myocardium [133,135], and demonstrate a role for altered m^6^A levels in modulating cardiac hypertrophy in mice [134,135]. Additionally, Kmietczyk et al. compared m^6^A-seq data with RNA sequencing and ribosome profiling data from cardiomyocyte specific ribosome tagged mice to correlate changes in m^6^A with genome-wide translational efficiency. Continued improvements in these genome-wide technologies along with longitudinal studies should provide a greater understanding of the complex proteomic signatures of cardiac disease progression.

## 6. Concluding Remarks

Evidence suggests a combination of inflammatory processes, myocyte apoptosis, fibrosis, and vascular rarefaction leads the transition to heart failure, yet the exact mechanisms governing the switch from adaptive to pathological hypertrophy remain to be determined. It is now known that in addition to transcriptional regulation, mRNA processing and modification regulate the proteome through dynamic translational control. Therefore events such as alternative splicing can determine the presence of *cis* regulatory elements in mRNA while alternative polyadenylation can also contribute to gene expression and protein diversity. Generation of such distinct mRNAs permissive or not to alternative translation initiation events, for example, can affect cardiac function. A great deal has also been discovered regarding upregulation of translational output in cardiac hypertrophy coming from studies in mice of mTOR function and its regulatory components. The recent recognition of RNA binding proteins mediating inflammatory gene translation in cardiac tissue, and the role of non-canonical translation in cardiac disease is providing additional targets for pursuing translational regulation therapeutically.
